# The Anti-Inflammatory Effects of Invigorating Kidney and Supplementing Qi Chinese Herbal Formulae in Asthma Patients

**DOI:** 10.1155/2017/3754145

**Published:** 2017-06-27

**Authors:** Lingwen Kong, Hongying Zhang, Yuxue Cao, Jingjing Le, Jinfeng Wu, Baojun Liu, Meixia Chen, Yijie Du, Jia Wang, Genfa Wang, Tao Yi, Xianmei Zhou, Gang Wang, Qing Miao, Suyun Li, Naiqing Zhao, Jingcheng Dong

**Affiliations:** ^1^Institute of Integrated Traditional Chinese and Western Medicine, Huashan Hospital Affiliated to Fudan University, Shanghai 200040, China; ^2^Department of Integrated Traditional Chinese and Western Medicine, Huashan Hospital Affiliated to Fudan University, Shanghai 200040, China; ^3^Pneumology Department, Jiangsu Provincial Hospital of TCM, Nanjing 210029, China; ^4^Pneumology Department, West China Hospital, Sichuan University, Chengdu 610041, China; ^5^Pneumology Department, Xiyuan Hospital of China Academy of Chinese Medical Sciences, Beijing 100091, China; ^6^Pneumology Department, The First Affiliated Hospital of Henan University of TCM, Zhengzhou City, Henan Province 450008, China; ^7^Department of Statistics, Fudan University, Shanghai 200032, China

## Abstract

**Background:**

The theories of Shen-reinforcement and Qi-supplementation are important in asthma treatment based on traditional Chinese medicine theories. Early studies suggested that Invigorating Kidney and Supplementing Qi herbal formulae, Bu Shen Fang Chuan (BSFC) and Bu Shen Yi Qi (BSYQ), conveyed promising results in asthma treatment. However, the efficacy and safety of the formulae need to be further investigated by a randomized double-blind clinical trial.

**Methods:**

328 eligible patients were randomly sent to BSFC, BSYQ, and placebo group. The two formulae were received as add-on therapy. The primary endpoints were rate of asthma exacerbation and Hamilton Rating Scale for Depression (HAM-D) score. The secondary endpoints included HPA axis function and inflammatory cytokine production profile. All indexes were measured before and after treatment.

**Results:**

The primary endpoints were not improved in both groups; however, the depression levels of subgroup patients with HAM-D score > 5 were improved in BSFC group. HPA axis functions and inflammatory cytokines level were also improved by two formulae. The incidences of adverse events were similar among groups.

**Conclusions:**

The two formulae had multiple advantage effects on neuroendocrine-immune system. They are worth used as a replacement therapy in asthma.

**Trial Registration:**

This trial is registered with clinical trial number ChiCTR-PRC-09000529.

## 1. Introduction

In traditional Chinese medicine (TCM) theory, “the essence of Kidney and Qi” is a core concept. If a patient has a “deficiency of Kidney and Qi,” his voice is weak or he does not want to talk; conversely, if a patient's voice is loud and powerful, the patient is full of Kidney and Qi. In addition, “chronic lung disease involving the Kidney” and “internal-external relations between the lung and Kidney” are also important theories in TCM, so the “Kidney and Qi deficiency” often occurs among asthmatic patients [1]. Studies by Shen show that “Kidney and Qi deficiency” is due to neuroendocrine disturbance and abnormal function of the hypothalamic-pituitary axis (HPA axis), which is consistent with the hypothesis that “the senile bell lies in hypothalamus” proposed by Everiff in the 1980s; therefore, asthmatics included in this study all suffered from long-term recurrence; meanwhile they were all with abnormal pathological changes in the anti-inflammatory system and HPA axis. In TCM, asthmatics suffering from these were diagnosed as “Kidney and Qi deficiency” and need to be treated with Invigorating Kidney and Supplementing Qi herbs to prevent exacerbation [1].

Asthma exacerbations cannot be completely prevented, despite receiving Global Initiative for Asthma (GINA) guideline-directed care [2, 3]. Psychosocial stress, such as depression or anxiety, is an important trigger in asthma exacerbation, which could deteriorate the HPA axis function and aggravate the airway's inflammatory response to environmental triggers [4–8]. An insufficient HPA axis function and an imbalance of Th1/Th2 all contribute to asthma exacerbation [9–11]. Supplementation of exogenous corticosteroids is one of the treatments available for the prevention of the exacerbation of asthma [12]. However, the treatments have been related to the suppression of the HPA axis and immune systems. TCM as a complementary and alternative medicinal modality is becoming popular for health care in Western countries. In recent years, increased numbers of double-blind, placebo-controlled small clinical studies investigating the efficacy and safety of TCM herbal products for asthma have been reported. These studies include ASHMI [13], Dingchuan Decoction [14], and STA-1 [15]. The researchers showed some evidence of clinical efficacy, including the normalization of HPA axis disturbances and antidepressant, anti-inflammatory, and immunomodulatory effects. This study is a further exploration in this area.

In this study, we used formulae of Bu Shen Fang Chuan (BSFC) and Bu Shen Yi Qi (BSYQ) as add-on treatments to prevent asthma exacerbation. The two formulae included main Tonifying Kidney and Qi herbs. These herbs have been used for thousands of years in Chinese medicine. BSFC formula has widely been used to treat asthma in the clinic in China. It has been claimed to be effective for preventing the exacerbation of asthma [16–18] and has been approved by the China Food and Drug Administration (Authorized Document Number: Z20103001 in Chinese medicine) for the treatment of seasonal onset asthma and chronic bronchitis. This formula includes Rehmannia Root, Epimedium Herb, Radix Rehmanniae Praeparata, South Dodder Seed, Malaytea Scurfpea Fruit, Common Yam Rhizome, Tangerine Peel, and Prepared Common Monkshood Daughter Root (shown in Table 1). The BSYQ formula is composed of both Tonifying Kidney herbs and Tonifying Qi herbs, so it also supplements Kidney and Qi deficiency. The formula includes Milkvetch Root, Epimedium Herb, and Rehmannia Root (shown in Table 2), which has shown satisfactory effects on chronic inflammatory airway disease for decades at the Huashan Hospital of Fudan University. Our studies have found that BSYQ can increase the lung function of COPD patients by regulating Th1/Th2 equilibrium and normalizing dysfunctions of the HPA axis [19, 20]. We also found that the Chinese herbs in the two formulae could improve the symptoms of asthmatic animals by enhancing the function of the HPA axis and regulating the inflammatory responses [21–24]. Previous study of our team further confirmed the association of depression, HPA axis function, and airway inflammation in inflammatory airway disease [25]. We found that the main components of the two formulae can promote adrenal gland weight gain and significantly restore plasma corticosterone and adrenocorticotropic hormone (ACTH) [26] and block LPS-induced airway inflammatory responses [27, 28]. Furthermore, the herbs can modify the social defeat-induced downregulation of the glucocorticoid receptor in mice [29]. On the basis of these findings, we undertook this randomized double-blind placebo-controlled parallel-group multicentre clinical trial to evaluate the efficacy and safety of these two formulae in asthma prevention.

## 2. Materials and Methods

The study was conducted in accordance with the guidelines of the Declaration of Helsinki and Tokyo for humans. It was approved by the Ethics Committee of Huashan Hospital Affiliated to Fudan University. The trial protocol, CONSORT checklist, and ethics approval document are available as supporting information. All patients provided written informed consent prior to their participation and the commencement of the study.

### 2.1. Participants

An asthma diagnosis was confirmed in accordance with the current guidelines of the Global Initiative for Asthma (GINA). The first subject was enrolled on November 11, 2009, and the last subject was enrolled on May 17, 2014; the experiment lasted 4 years and 6 months. All patients included in the study had at least one attack history of asthma in the last three years. During run-in period, they only inhaled short-acting *β*2 agonists or short-acting *β*2 agonists/corticosteroids and only those with a stable condition (ACT ≥ 20 score, FEV1% ≥ 60%) can be included. If the included patient showed one of the following, it would be defined as a deterioration and the patient must withdraw from the trial: (1) an increased rescue short-acting *β*2-agonist (≥10 puffs/day) for at least 2 consecutive days; (2) hospitalization; (3) emergency room visit; (4) need for systemic corticosteroids for at least 3 days; and (5) a 15% decrease in morning peak expiratory flow (PEF) or a 20% decline in clinic FEV1. The exclusion and inclusion criteria are shown in Table 3.

### 2.2. Study Design

This was a 12-month, randomized double-blind placebo-controlled parallel-group multicentre study (http://www.chictr.org, clinical trial number: ChiCTR-PRC-09000529). Participants were enrolled at five trial sites in five cities in mainland China. The allocations of the subjects at each centre are shown in Supplementary Table 1 (see Supplementary Material available online at https://doi.org/10.1155/2017/3754145).

### 2.3. Randomization and Masking

Randomization was performed with SAS 8.2. Eligible patients were assigned a randomized number according to a predetermined list at each centre. These numbers were assigned to patients in sequential order and registered in the patient enrolment list. The group assignments were concealed. Neither the evaluators nor the on-call admitting team were made aware of the actual treatment group assignments. Emergency envelopes containing the randomization code were provided to the investigators and were examined at the end of the trial to ensure that the blinded conditions had been maintained. The eligible patients were stratified by lung function (FEV1% ≥ 80% or FEV1% 60~79%) and the 5 research centres.

### 2.4. Procedures

Eligible asthma patients were divided into 2 categories.

(1) Patients who were treated with inhaled corticosteroids/short-acting *β*2 agonist (current maintenance treatment) initially underwent a 2-month screening period. During this time, patients were only treated with inhaled corticosteroids/short-acting *β*2 agonist.

(2) Patients who were only treated with short-acting *β*2 agonist (current maintenance treatment) initially underwent a 2-week run-in period. During this time, patients were only treated with inhaled short-acting *β*2 agonist.

In run-in period, any participants who showed an exacerbation of symptom requiring additional medications were excluded from the study. If they did not show the acute exacerbation symptoms, they were randomly assigned (in a 1 : 1 : 1 ratio) to BSFC group, BSYQ group, and placebo group. In the first 6 months of trial, they inhaled corticosteroids and/or short-acting *β*2 agonist add-on BSFC, BSYQ, or placebo; in the last 6 months, they stopped add-on treatment drugs and followed up for 6 months.

After the screening assessment, participants entered the study and their visits were scheduled. Participants attended all 6 visits over 12 months. Their 4 visits received an add-on treatment with BSFC, BSYQ, or placebo for 6 months, and then they were followed up 2 visits without add-on treatment for 6 months. At visit 1, a baseline assessment was performed and the clinical history was acquired. At visit 6, we assessed the time to next asthma exacerbation. The assessments of effect and safety were made at visit 3 and visit 4. At each visit, patients were required to take any remaining investigated drugs to the researcher. We counted the drugs that patient has taken during the past visit and then recorded and calculated adherence.

During the visits 1 to 4, patients received an add-on treatment with two formulae or placebo. The subjects in the BSFC (109 patients) group received oral BSFC tablets (5 tablets (0.25 g/tablet) 3 times per day (tid)). Subjects in the BSYQ group (112 patients) received oral BSYQ granules (5.5 g (5.5 g/bag) twice a day (bid)). The subjects in the placebo group (107 patients) received oral placebo tablets and placebo granules. These study drugs were supplied in a kit labelled with a unique code number. Randomization codes were concealed from all of the staff members at the investigational sites and from the staff members of the sponsor who had access to the site information and patient data.

The BSFC and placebo tablets were produced by Taiji Pharmaceutical Technologies Co., Ltd. (batch numbers 10100006, 10100007, and 10100008). Each BSFC tablet contained 0.25 g of dried aqueous extract. The total daily dose of BSFC (3.75 g) was equivalent to extracts of a mixture of the raw herbs Radix Rehmanniae Praeparata (10 g), Epimedium Herb (10 g), Rehmannia Root (10 g), South Dodder Seed (10 g), Malaytea Scurfpea Fruit (10 g), Common Yam Rhizome (10 g), Tangerine Peel (3 g), and Prepared Common Monkshood Daughter Root (6 g).

The BSYQ and placebo granules were produced by Sichuan Jiangyin Tianjiang Pharmaceutical Technologies Co., Ltd. (batch numbers 1006380, 1006381, and 1006382). Each bag of BSYQ granules contained 5.5 g of dried aqueous extract. The total daily dose of 1 bag of BSYQ (5.5 g) was equivalent to extracts of a mixture of raw Radix Astragali Root (30 g), Epimedium Herb (20 g), and Rehmannia Root (15 g).

Every herb used in the two formulae followed the standard in Chinese pharmacopoeia; for example, the content of icarrin in Herba Epimedii must be >0.5% according to pharmacopoeia. The qualitative and quantitative analysis of the chemical constituents in the two formulae was performed by HPLC-MS and HPLC-UV methods (see Supplementary Material). Agilent Technologies Masshunter Workstation Acquisition Software (Rev. B. 05. 01) (LC/TOF) was used for the data acquisition. In addition, Q-TOF Agilent Technologies Masshunter Workstation Quantitative Analysis Software (Version B. 05. 02) was also used for data analysis [30]. All of the reference standards for BSFC and BSYQ were purchased from Chengdu Pusi Bio-Technology (Chengdu, China). The purities of all of the compounds were above 98% based on HPLC-UV. HPLC analysis was completed using an Agilent 1260 series (Agilent, Waldbronn, Germany) coupled with electrospray ionization (ESI) and a photodiode array detector. Chromatographic separation was performed on Agilent Poroshell 120-C18 columns (2.7 × 100 mm, 2.7 *μ*m) at 35°C. The mobile phase was a mixture of water containing 0.1% formic acid and acetonitrile. The gradient elution protocols for BSFC tablets and BSYQ granules are presented in sTables 2 and 3. The flow rate was 0.35 ml/min, and the injection volume was 3 *μ*L. The MS parameters were as follows: flow rate of drying gas (nitrogen) 10 L/min, nebulizer pressure 30 psig, drying gas temperature (nitrogen) 350°C, and capillary voltage 3500 V. The detection wave length for BSFC was 254 nm. For details, please see our researches [19, 20, 23, 30, 31].

For the duration of the study, the recordings of peak expiratory flow (PEF) were performed each morning by the patients before the medication was inhaled; leukotriene modifiers, antihistamines, intravenous or oral glucocorticoids, and other TCMs were prohibited. At the end of the sixth month of the trial, the add-on treatment was stopped and all patients continued to be followed for another 6 months. Subjects who required additional intervention at any time because of disease severity were withdrawn from the study.

### 2.5. Study Outcomes

The primary endpoints were the rate of asthma exacerbation and the Hamilton depression rating scale (HAM-D). Exacerbations were defined as a deterioration in asthma leading to at least one of the following: (1) an increased rescue short-acting *β*2-agonist (≥10 puffs/day) for at least 2 consecutive days; (2) hospitalization; (3) emergency room visit; (4) need for systemic corticosteroids for at least 3 days; and (5) a 15% decrease in morning PEF or a 20% decline in clinic FEV1 [32]. Other indexes included 8:00 am morning plasma cortisol; ACTH; CRH; serum IL-2, IL-4, IL-5, IL-6, IL-10, IL-13, and IL-17; IFN-*γ*; TNF-*α*; eosinophil cationic protein (ECP); IgE; and the eosinophil counts in blood. All indexes were evaluated before and after 6 months of treatment.

### 2.6. Safety Assessments

The adverse responses (AR)/adverse events (AE) were written daily with a record of the occurrence time, duration, frequency, treatment, and result of treatment. The grading of these adverse events followed the norms of the World Health Organization Recommendations for Grading of Acute and Subacute Toxicity [33]. Investigators were asked to rate the likelihood of a connection between the study drug and the AR/AE as 0 (unlikely), 1 (possible), or 2 (probable). Haematology, urine, and serum chemistry testing and electrocardiograms were performed before and after 6 months of treatment.

### 2.7. Statistical Analysis

The analysis of efficacy was performed with the intention-to-treat (ITT) population, which included all of the eligible patients who received at least one dose of medication and who had at least a baseline and one postbaseline assessment. Variables that were unusually distributed were log-transformed to an approximate normal distribution for analysis. The results are explained as the mean ± standard deviation or median, unless otherwise stated. The baseline characteristics of the subjects in those relevant groups were assessed by using one-way analysis of variance (ANOVA) for continuous variables and the *χ*^2^ test for categorical variables. Following analysis of variance, Fisher's least significant difference test was used to further explore and compare the mean of one group with the mean of another. The analysis of covariance was used for controlling potential confounding variables.

For the primary endpoint, log rank tests were completed to analyse the cumulative exacerbation in those three groups. Categorical variables, including patient treatment compliance, safety, and rate of dropping-out, were analysed with the Chi-square or Fisher's exact test, as appropriate, to test differences in proportions. One-way variance analysis was performed to detect the differences among continuous variables, including inflammatory cytokines, eosinophil counts, and eosinophil cationic protein (ECP). If the differences were significant, a LSD *t*-test would be used to measure the difference between the two groups.

The analyses of safety were made from the tests on patients who had at least one evaluation of safety after treatment of drugs (Safety Set). Pearson's Chi-square test or Fisher's exact test was used to detect the differences among the three groups. All analyses for baseline, treatment effects, and safety assessment were performed by using SAS 9.13 software (SAS Institute Inc., Cary, NC, USA), *P* < 0.05 was considered statistically significant, and all tests were 2-tailed.

## 3. Results

**Figure 1:**
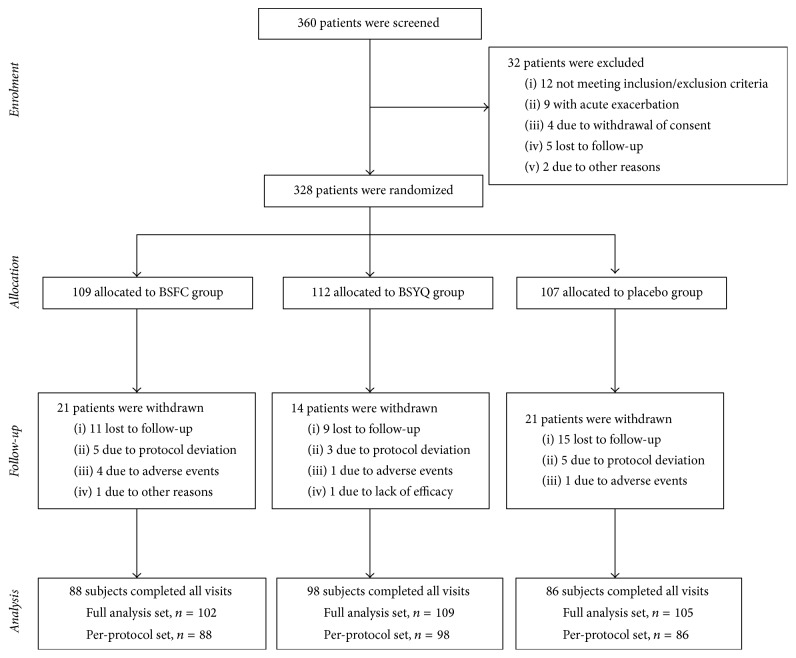
Number of patients who were enrolled and assigned to a study group and who completed the study. Subjects were recruited by respiratory physicians at the five participating clinical centres.

Five research centres in China were included in this study. Of 360 screened subjects, 328 were randomized; the ITT population was composed of 317 patients, and the PP population was composed of 272 patients. Subjects were randomly assigned to the BSFC, BSYQ, or placebo group. The study design is shown in Figure 1.

### 3.1. Baseline Characteristics

The 3 treatment groups were well balanced in terms of age, weight, height, history of asthma, life events, family history of allergic diseases, chest X-ray, lung function test, HAM-D score, and ACT score (see Table 4). There were more women in the BSFC group than in the BSYQ and placebo groups (73.5%, 63.0%, and 49.0%, respectively; *P* = 0.002). The adherence of patients during the trial in all groups exceeded 95% (98.0% for BSFC versus 98.2% for BSYQ versus 93.3% for placebo). There was no significant differentiation among groups (*P* = 0.111).

### 3.2. Primary Outcomes

#### 3.2.1. The Rate of Acute Exacerbations

**Figure 2:**
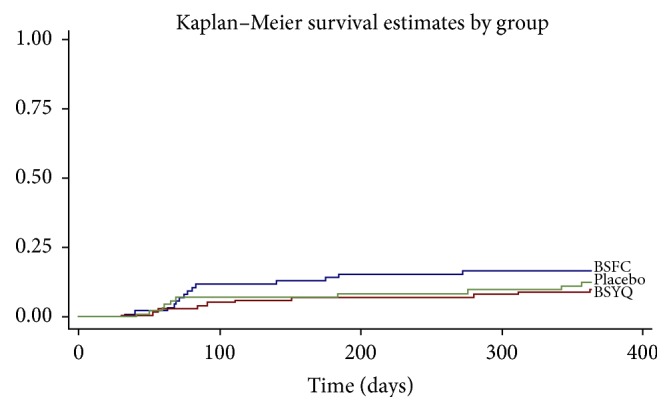
The Kaplan–Meier curves for time of first severe asthma exacerbation. The incidence rates (%) of BSFC, BSYQ, and placebo group were 5.26%, 3.07%, and 3.74%, respectively; *P* = 0.434.

Neither BSYQ nor BSFC treatment significantly reduced the rate of acute exacerbations after treatment of 11 months. All 14 patients experienced acute exacerbation in the BSFC group, 10 patients in the BSYQ group, and 10 patients in the placebo group. Estimates of the reexacerbation rates (%) were 0.05262, 0.03072, and 0.03747, respectively (*P* = 0.434, Figure 2).

#### 3.2.2. Effect of Formulae Treatment on the HAM-D Score

**Figure 3:**
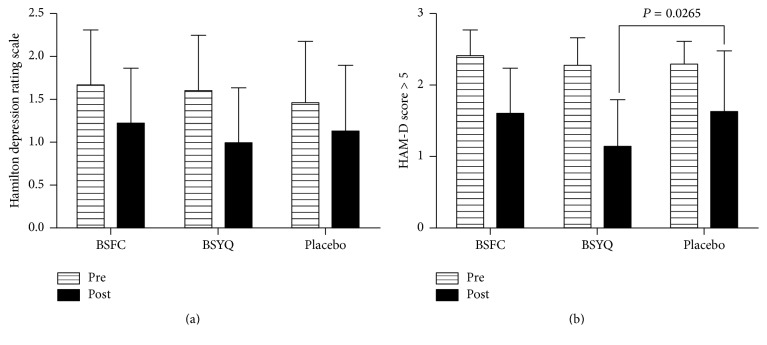
Effect of treatment with the two formulae on the 24-item Hamilton depression rating scale. (a) The HAM-D score (log-transformed) was evaluated before and after treatment, and a greater reduction from baseline was found in the two formula groups. However, the difference was not significant for three groups (*F* = 1.608, *P* = 0.2026). (b) The subgroup of patients with a HAM-D score > 5 (log-transformed) was evaluated before and 6 months after treatment, with the BSYQ group (*N* = 36) showing the greatest reduction after treatment compared with placebo (*N* = 24, *P* = 0.0265).

The HAM-D scores were not significantly different among the BSFC, BSYQ, and placebo groups (*P* = 0.2026), although treatment with BSFC and BSYQ had a greater reduction from baseline than the placebo after 6 months of treatment. Significantly reduced HAM-D scores from baseline were found in the subgroup with a HAM-D score > 5 of the BSYQ group compared with placebo (*P* = 0.0265, see Figure 3).

### 3.3. Secondary Outcomes

#### 3.3.1. Effect of Formulae Treatment on HPA Axis Expression

**Figure 4:**
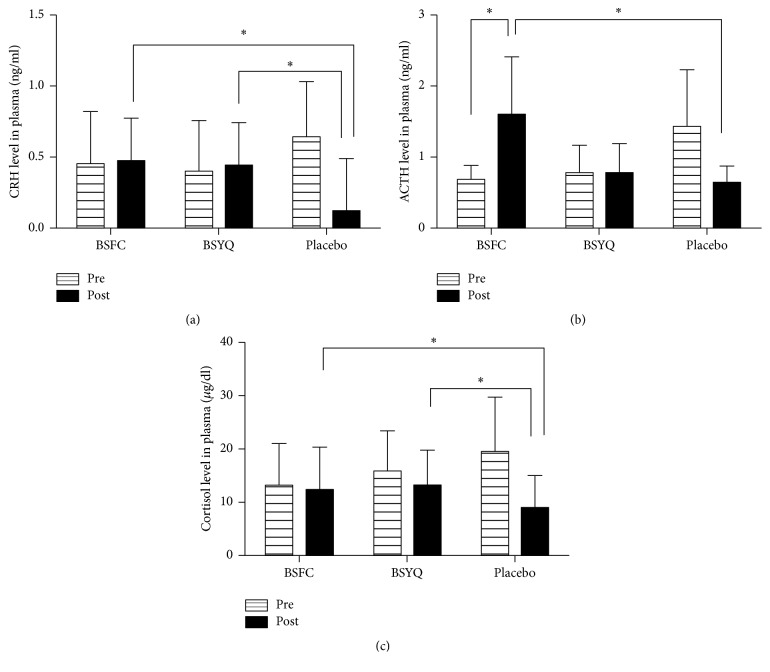
Effects of 6 months of treatment with the two formulae or placebo on the HPA axis. (a) CRH, (b) ACTH, and (c) cortisol levels (7:30–8:30 AM) were determined by ELISA. Venous blood samples were obtained from all patients before and 6 months after treatment. Plasma CRH, ACTH, and cortisol levels were measured using commercial ELISA kits (Phoenix Pharmaceuticals, Inc., Burlingame, CA, USA) according to the manufacturer's instructions. The CRH data were log-transformed, and all data are the means ± SDs for the variable with unbalanced baseline using the baseline correction in the analysis of covariance. ^*∗*^*P* < 0.05.

As shown in Table 5 and Figure 4, before the treatment, the CRH, ACTH, and cortisol in placebo group were all higher than two formulae groups; however, after treatment CRH levels were significantly reduced from baseline in placebo group and increased in both treatment groups, (*P* = 0.049, see Table 5 and Figure 4). The plasma levels of ACTH were also increased in both BSFC and BSYQ groups, while the ACTH level was significantly reduced in the placebo group after treatment and the difference between the BSFC and placebo groups was significant (*P* < 0.05, see Table 5 and Figure 4). In the same way, the cortisol concentrations were also significantly changed from baseline compared with placebo group (*P* < 0.05, see Table 5 and Figure 4).

#### 3.3.2. Effect of the Formulae Treatment on the Inflammatory Cytokines Profiles

**Figure 5:**
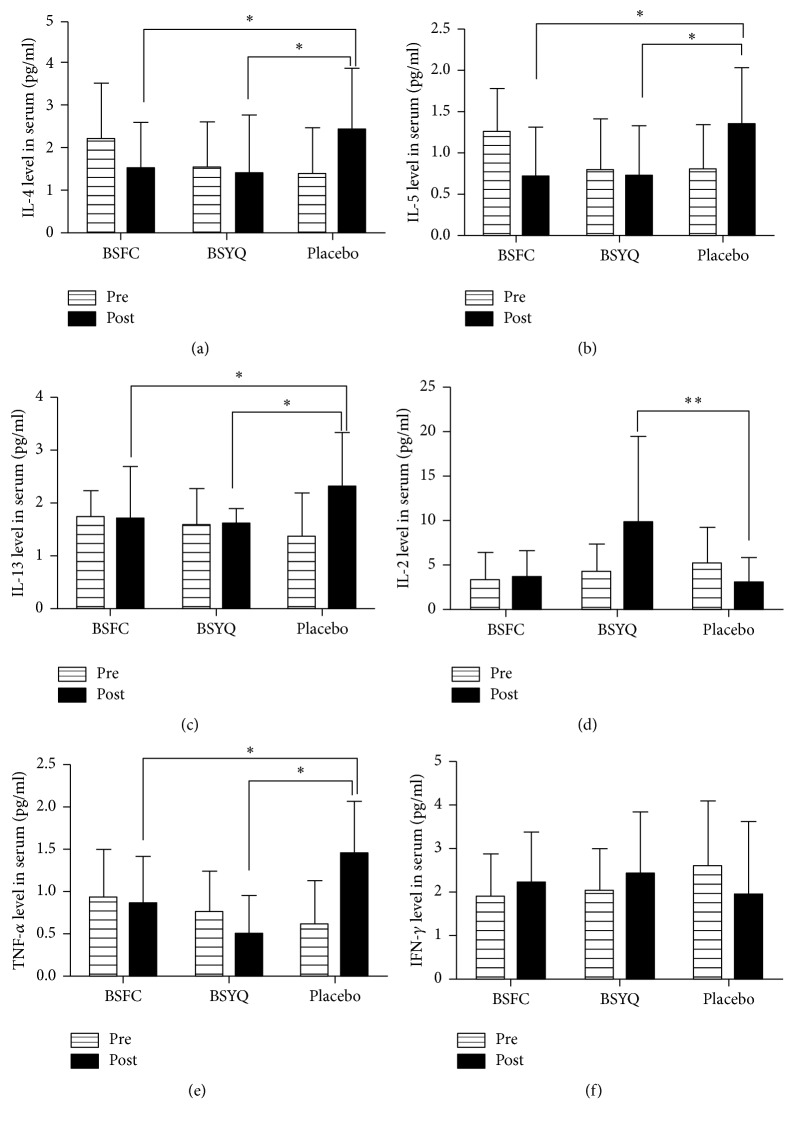
Effect of 6 months of treatment with the two formulae on serum inflammatory cytokines. Serum IL-4 (a), IL-5 (b), IL-13 (c), IL-2 (d), TNF-*α* (e), and IFN-*γ* (f) levels were determined by ELISA. Venous blood samples were obtained from all patients before and 6 months after treatment. Serum IL-2, 4, 5, 13, TNF-*α*, and IFN-*γ* were measured using commercial ELISA kits (Huntington Station, NY, USA) according to the manufacturer's instructions. Data are means ± SDs. The IL-4, TNF-*α*, and IFN-*γ* data were log-transformed. ^*∗*^*P* < 0.05; ^*∗∗*^*P* < 0.01.

As shown in Figure 5, serum IL-5, IL-4, and IL-13 were reduced from baseline in both the BSFC and BSYQ groups compared to the placebo group (*P* < 0.05). In contrast, IL-2 was significantly upregulated in the BSYQ group (*P* < 0.01), slightly increased in the BSFC group, and significantly reduced in the placebo group. There were significant differences among the three groups (*P* = 0.0046, Figure 5). Interferon-gamma (IFN-*γ*) was also increased in both formulae groups, while it was significantly reduced in placebo. The BSYQ group had the highest increased level of IFN-*γ* (*P* = 0.06, Figure 5). Moreover, proinflammatory cytokine-TNF-*α* was significantly blocked by both formulae and the differences were significant among the three groups (*P* = 0.006, Figure 5). IL-6 was also blocked in the BSYQ group; on the contrary it was significantly increased in the placebo group; however, the difference was not significant (*P* = 0.1363, see sFigure 1). The changes for IL-10 and IL-17A were also not significantly different among the three groups (*P* > 0.05, see sFigure 1). The effects of BSFC or BSYQ treatment on the eosinophil counts and IgE and ECP expression are shown in Supplementary sFigure 1.

### 3.4. The Main Compounds of BSFC and BSYQ

There were 12 and 16 main active chemical compounds detected in BSFC and BSYQ, respectively. The typical total ion chromatography of BSFC and BSYQ and the UV chromatography of BSFC are shown in Supplementary Material (sFigure 2). Errors were calculated by analysing 3 batches of BSFC and BSYQ.

### 3.5. Safety

A total of 317 patients were recruited and integrated in the Safety Set. The frequency of adverse events (AE)/responses (AR) associated with treatment in the BSFC, BSYQ, and placebo groups was 15/15, 15/15, and 13/15, respectively. There were no significant differences in either the frequency of AE/AR among the three groups or the occurrence of serious adverse events (see sTable 4 and sTable 5). In the study, the most frequent AE reported were discomfort of the stomach, rash, and low back pain. One subject in the BSFC group reported having abnormal hyperglycaemia, but it is not related to the treatment. A total of 3 patients were found to have abnormal liver function after treatment: 1 subject was in placebo group and 2 subjects displayed minimally abnormal liver function tests before add-on treatment. No subject was found with any renal function abnormal. Haematology, urine, and electrocardiograms were not reported as abnormal after treatment in any patients.

## 4. Discussion

There is increasing scientific evidence supporting the use of TCM for asthma treatment [34]. In this clinical study, we measured the anti-inflammatory effects and safety of two antiasthma formulae. The samples not only were relatively large in size but also covered different regions of north and south China. Moreover, our study had three groups with double-placebo medications, making it more unbiased than other studies.

Compared with the changes of inflammatory cytokines and HPA axis elements, the result of acute exacerbation was not improved after treatment. The reasons of this result were various. Firstly, the 6 months of treatment may be too short to see a significant effect in the index; in general the improvement of HPA axis function and the control of the chronic inflammation can lead to a reduction of acute exacerbation in the asthmatic patients. In addition, the more sensitive research method was also needed for detecting preventive effects to obtain more satisfactory results. Lastly, a higher number of patients were withdrawn at visit 4 which was contributed for the unsatisfactory results.

In this study, we used Hamilton's original scale (HAM-D) as an indication of depression. The results of the HAM-D score > 5 demonstrated that the two formulae reduced the depression level of patient, especially in the BSYQ group. Psychological stress has been regarded as an important trigger in atopic diseases [9], and both animal and clinical studies confirmed that exogenously applied stress or stressful life events could increase allergen-induced airway inflammation [35] and the risk of a new asthma attack [36]. The emphasis of TCM is to treat patients as a whole [37]; actually, in the study the two formulae showed multiple beneficial effects both in mind and in body, because we also found HPA axis function was improved and the Th1/Th2 response was reversed significantly in addition to the antidepression effect. These kinds of multiple benefits on the NEI system have been also reported by previous research [23, 38]. Our results indicated that the upregulation of TNF-*α* and IL-6 was suppressed by the two formulae, which also confirmed our previous research [27, 28]. Proinflammatory cytokines, such as TNF-a, IL-1, and IL-6, are the major mediators between the brain, HPA axis, and immune system and continuous prolonged upregulation of proinflammatory cytokines can lead to depression and HPA axis suppression, which may trigger exacerbation of asthma [39–41].

All herbs in BSFC and BSYQ have a long history of human use in China, and they are considered to be safe if they are used in accordance with the TCM's principles, either alone or in formulae. Both formulae tested in the current study do not contain “Ma Huang” or “*Datura metel*.” “Ma Huang” is a classic antiasthma herb and a source of ephedrine that can relieve bronchial spasm and reduce the secretion of mucus, while “*Datura metel*” is known to be an anticholinergic herb that reduces spasms by blocking the transmission of nerve impulses. Both herbs relieve the symptoms of asthma rapidly but have some adverse effects in the cardiovascular and central nervous systems. Fortunately, the effects of BSFC and BSYQ on asthmatics were based on improving HPA axis function, regulating disordered immunomodulatory effects, and reducing depression levels; thus a longer regulated period was needed to observe obvious improvement in clinical symptoms.

We detected the primary bioactive compounds of the two formulae. We found that BSYQ not only contains fundamental compounds of “Nourishing Kidney” herbs, such as Epimedin A, Epimedin B, Epimedin C, and Baohuoside-I, but also contains Astragaloside I, Astragaloside II, and Astragaloside IV, which are constituents of “Replenishing Qi” herbs. In addition, psoralenoside, isopsoralenoside, coryfolin, psoralen, and corylifolinin were primarily detected in the BSFC, compounds that BSYQ did not include. Thus, the difference in herbal compounds may explain why BSFC was the better treatment for improving the HPA axis function, while BSYQ had better anti-inflammatory, immunomodulatory, and antidepression effects. We would anticipate conducting a separate but more extensive experiment on the effects of these compounds in the BSFC and BSYQ. We have performed power calculations for this study to show statistical powers ranging from 39%–76% due to variance in difference of means. There may be more true positive results in an extended sample in near further.

This study has shown that long-term treatment with two formulae was safe given that the frequency of adverse events/responses was not different from placebo. No severe adverse events or death was reported. No clinically relevant treatment differences in vital signs or liver and renal function parameters were observed among groups.

The limitations in this study deserve comment. The HPA axis function and the immune system disorder were all improved after treatment and the depression level was decreased compared with placebo. However, the clinical indicators of the rate of asthma exacerbations were not changed, which suggests that the 6-month follow-up and 6-month treatment period may not be long enough and that a number of promising results were not significantly different in our study. In addition, the more sensitive experimental method needs to be used to confirm the changes of HAM-D scores; meanwhile, more scientific design should be conducted in future research. Although the adherence of patients during the trial in all groups exceeded 95%, a higher number of patients were withdrawn at visit 4 which was one of the reasons for the unsatisfactory results and the more stringent management needs to be performed in further research.

In conclusion, the two formulae did not reduce the rate of acute exacerbation; however, they showed multiple beneficial effects on NEI system: they reduced psychological stress and improved HPA axis function, they modulated Th1/Th2 balance and suppressed proinflammatory cytokines, and they were safe and well tolerated. BSFC made better treatment in improving the HPA axis function, while BSYQ had better antidepression and immunomodulatory effects. They appeared safe and well tolerated in the study.

## Supplementary Material

1. sTable 1. Allocation of subject in each center.
2. sTable 2. The gradient elution for quantitative analysis of BSFC.
3. sTable 3. The gradient elution for quantitative analysis of BSYQ.
4. sTable 4. Adverse events.
5. sTable 5. Adverse responses.
6. sFigure 1. The main compounds of BSFC and BSYQ.
7. sFigure 2. Effect of the Formulae Treatment on serum (A) IL-6, (B) IL-10, (C) IL-17, (D) IgE, (E) ECP and (F) eosinophil numbers.

## Figures and Tables

**Table 1:** Component herbs of the BSFC formula.

Pharmaceutical name	Botanical plant name	Family and plant part used	English name	Chinese name	Daily doses
Herba Epimedii	*Epimedium brevicornum* Maxim.	Berberidaceae; aerial part	Epimedium Herb	Yin Yang Huo	10 g
Rehmanniae Radix	*Rehmannia glutinosa* Libosch.	Scrophulariaceae; root tuber	Rehmannia Root	Di Huang	10 g
Rehmanniae Radix praeparata	*Rehmannia glutinosa* Libosch.	Scrophulariaceae; root tuber	Radix Rehmanniae Praeparata	Shu Di Huang	10 g
Cuscutae semen	*Cuscuta chinensis* Lam	Convolvulaceae; semen	South Dodder Seed	Tu Si Zi	10 g
Psoraleae Fructus	*Psoralea corylifolia* L.	Leguminosae; fructus	Malaytea Scurfpea Fruit	Bu Gu Zhi	10 g
Dioscorea opposita Rhizome	*Dioscorea opposita* Thunb	Dioscoreaceae; rhizome	Common Yam Rhizome	Shan Yao	10 g
Citri Reticulatae Pericarpium	*Citrus reticulata* Blanco	Rutaceae Pericarpium	Tangerine Peel	Chen Pi	3 g
Aconiti lateralis radix praeparata	*Aconitum carmichaelii* Debx	Ranunculaceae radix praeparata	Prepared Common Monkshood Daughter Root	Shu Fu Zi	6 g

**Table 2:** Component herbs of the BSYQ formula.

Pharmaceutical name	Botanical plant name	Family and plant part used	English name	Chinese name	Daily doses
Radix Astragali	*Astragalus membranaceus* (Fisch.) Bunge.	Leguminosae; root	Milkvetch Root	Huang Qi	30 g
Herba Epimedii	*Epimedium brevicornum* Maxim.	Berberidaceae; aerial part	Epimedium Herb	Yin Yang Huo	20 g
Rehmanniae Radix	*Rehmannia glutinosa* Libosch.	Scrophulariaceae; root tuber	Rehmannia Root	Di Huang	15 g

**Table 3:** Exclusion and inclusion criteria.

Exclusion criteria	(1) With a history of pulmonary embolism, pulmonary fibrosis, chronic obstructive pulmonary disease, pleural effusion, active tuberculosis, bronchiectasis, and pneumonectomy
	(2) Pregnant and lactating women
	(3) Showing exacerbation of symptoms requiring additional medications during screening period
	(4) Malignant tumour and blood disease
	(5) Participating in other clinical trials in the past three months
	(6) Use of oral corticosteroids in the previous four weeks
	(7) Experienced an infectious disease within four weeks
	(8) With heart, liver, kidney, or other organ diseases

Inclusion criteria	(1) 18 to 70 years old; any gender
	(2) With mild-to-moderate asthma
	(3) ACT ≥ 20 score, FEV1% ≥ 60%
	(4) Having a history of recurrent asthma exacerbations in the past two years
	(5) Without upper and lower respiratory tract infections in the past four weeks
	(6) With Kidney and Qi deficiency based on TCM syndrome differentiation
	(7) Understanding of the research protocol and consent to participate

**Table 4:** Patient characteristics and demographics.

	BSFC	BSYQ	Placebo	*P* value
*Mean age, years (SD)*	47.43 (12.68)	46.69 (12.87)	46.64 (13.18)	0.88
*Sex, n (%)*				0.002
Male	27 (26.47)	40 (37.04)	53 (50.96)	
Female	75 (73.53)	68 (62.96)	51 (49.04)	
*High, cm, mean (SD)*	161.32 (7.89)	162.82 (8.11)	163.58 (7.29)	0.1
*Weight, kg, mean (SD)*	62.27 (10.31)	62.63 (10.19)	62.85 (11.04)	0.95
*The number of asthmatic attacks in the last two years*				
First year, mean (SD)	1.72 (0.87)	1.61 (0.76)	1.77 (0.87)	0.33
Second year, mean (SD)	1.68 (0.88)	1.50 (0.56)	1.52 (0.79)	0.07
*FEV1/FVC, predicted*				
FEV1 (%), mean (SD)	80.72 (18.72)	8.53 (20.31)	79.11 (18.26)	0.73
Mean (SD)	76.77 (13.39)	75.48 (16.56)	75.17 (14.33)	0.6
*History of asthma*				0.42
Yes, *n* (%)	22 (21.57)	28 (25.93)	30 (28.85)	
No, *n* (%)	80 (78.43)	80 (74.07)	74 (71.15)	
*Family history of allergic diseases*				0.52
Yes, *n* (%)	71 (69.61)	72 (66.67)	65 (62.50)	
No, *n* (%)	31 (30.39)	36 (33.33)	39 (37.50)	
*Family history of eczema*				0.38
Yes, *n* (%)	0 (0.00)	1 (0.93)	0 (0.00)	
No, *n* (%)	102 (100.0)	107 (99.07)	104 (100.0)	
*Family history of allergic rhinitis*				0.9
Yes, *n* (%)	8 (7.84)	9 (8.33)	7 (6.73)	
No, *n* (%)	94 (92.16)	99 (91.67)	97 (93.27)	
*Life events*				0.36
Yes, *n* (%)	0 (0.00)	1 (0.98)	0 (0.00)	
No, *n* (%)	96 (100)	101 (99.02)	90 (100)	
*Chest X-ray*				0.56
Normal, *n* (%)	74 (79.57)	84 (81.55)	71 (74.74)	
Abnormal, *n* (%)	19 (20.43)	19 (18.45)	24 (25.26)	

**Table 5:** Effect of formulae treatment on HPA axis function.

Variables	Per-protocol set				
	BSFC	BSYQ	Placebo	*F*	*P* value
	*n* = 88	*n* = 98	*n* = 86		
CRH (logarithm)					
Before treatment	0.45 ± 0.57	0.40 ± 0.55	0.64 ± 0.59	2.954	0.0548
Min–max	−1.27~1.85	−1.89~1.30	−1.00~2.02		
After treatment	0.47 ± 0.60	0.44 ± 0.70	0.12 ± 0.87	3.101	0.0492
Min–max	−0.79~1.81	−1.55~1.64	−2.58~1.87		
ACTH					
Before treatment	0.68 ± 0.20	0.77 ± 0.70	1.43 ± 1.95	9.556	0.001
Min–max	0.12~1.28	0.08~6.89	0.27~6.96		
After treatment	1.59 ± 1.88	0.78 ± 0.41	0.64 ± 0.23	5.274	0.0069
Min–max	0.40~6.53	0.42~1.99	0.13~1.21		
CORT					
Before treatment	13.09 ± 7.64	15.72 ± 7.76	19.51 ± 10.20	8.726	0.0002
Min–max	0.20~45.95	1.23~52.87	6.65~49.68		
After treatment	12.24 ± 8.11	13.09 ± 6.90	8.93 ± 5.99	3.989	0.0222
Min–max	1.64~29.45	3.68~36.43	0.23~22.22		
