# Supplementary material for: The Anti-Inflammatory Effects of Invigorating Kidney and Supplementing Qi Chinese Herbal Formulae in Asthma Patients
Source: Evid Based Complement Alternat Med. 2017 Jun 27;2017:3754145. doi: 10.1155/2017/3754145 (PMC5504930; doi:10.1155/2017/3754145)
Supplement: Supplementary file 1 — 1. sTable 1. Allocation of subject in each center. 2. sTable 2. The gradient elution for quantitative analysis of BSFC. 3. sTable 3. The gradient elution for quantitative analysis of BSYQ. 4. sTable 4. Adverse events. 5. sTable 5. Adverse responses. 6. sFigure 1. The main compounds of BSFC and BSYQ. 7. sFigure 2. Effect of the Formulae Treatment on serum (A) IL-6, (B) IL-10, (C) IL-17, (D) IgE, (E) ECP and (F) eosinophil numbers. [file 3754145.f1.pdf]

Supplementary Material Catalogue

Part 1 sTables

sTable1. Allocation of Subject in Each Center

sTable 2. The gradient elution for quantitative analysis of BSFC

sTable 3. The gradient elution for quantitative analysis of BSYQ

sTable4 . Adverse events

sTable 5. Adverse responses

Part 2 sFigures

sFigure 1. The main compounds of BSFC and BSYQ

sFigure 2. Effects of 6 months of treatment with two formulae or placebo on serum (A) IL-6, (B)IL-10, (C) IL-17, (D) IgE , (E) ECP and (F) eosinophil numbers.

Part 1 sTables

sTable1 Allocation of Subject in Each Centre

	Huashan Hospital	Xiyuan Hospital CACMS	West China Hospital of Sichuan University	The First Affiliated Hospital of Henan University of TCM	Jiangsu Province Hospital of TCM
Treatment Group (Subject)	40	20	20	20	20
Treatment Group (Subject)	40	20	20	20	20
Control Group (Subject)	40	20	20	20	20
Total: 360	120	60	60	60	60

sTable 2. The gradient elution for quantitative analysis of BSFC

Time（min）	0.1% Formic acid water（%）	Acetonitrile（%）
0	87	13
1	87	13
2.5	70	30
5	50	50
7	20	80
7.5	10	90
10	10	90
10.5	87	13
15	87	13

sTable 3. The gradient elution for quantitative analysis of BSYQ

Time（min）	0.1% Formic acid water（%）	Acetonitrile（%）
0	92	8
8	74	26
16	65	35
23	10	90
26	10	90

sTable4 . Adverse events

Adverse events	BSFC	BSYQ	Placebo	F	P
				Chi-square	P-Value
N(missing)	103(0)	109(0)	105(0)	0.43	0.8066
Frequency of adverse events	25	24	21		
rate（%）	24.27	22.02	20.00		
				Chi-square	P-Value
N(missing)	103(0)	109(0)	105(0)	0.2167	0.897
Patients with adverse events (%)	15(14.56)	15(13.76)	13(12.38)		
Patients without adverse events (%)	88(85.44)	94(86.24)	92(87.62)		

sTable 5.　Adverse responses

Adverse responses	BSFC	BSYQ	Placebo	F	P
				Chi-square	P-Value
N(missing)	103(0)	109(0)	105(0)	0.57	0.7518
Frequency	24	21	20		
Rate（%）	23.30	19.27	19.05		
				Chi-square	P-Value
N(missing)	103(0)	109(0)	105(0)	0.029	0.986
Patients with adverse response (%)	15(14.56)	15(13.76)	15(14.29)		
Patients without adverse response (%)	88(85.44)	94(86.24)	90(85.71)		

Part 2 sFigures

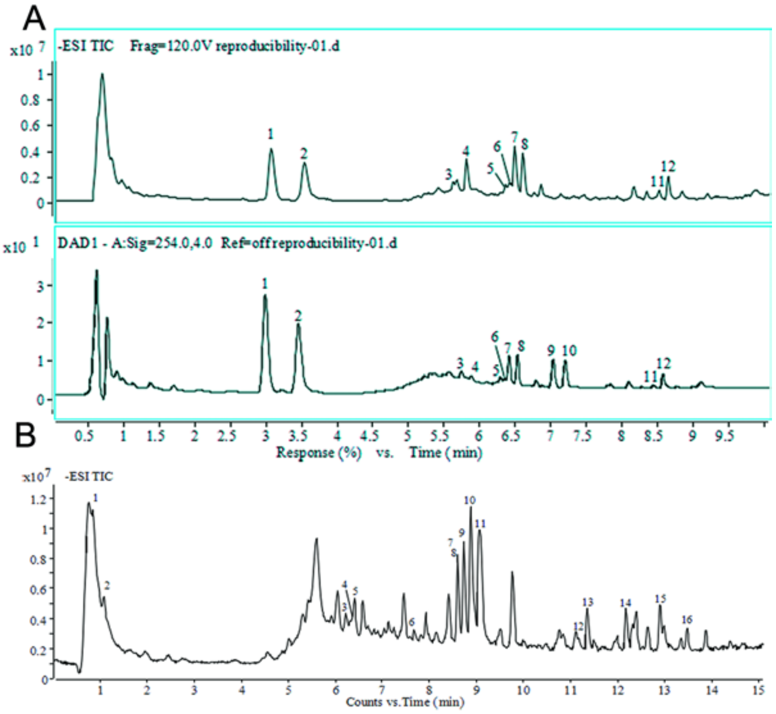

sFigure 1. (A) Total ion chromatography (above) and UV chromatography (below) of the BSFC formula(Formula A). (B) Total ion chromatography of the BSYQ formula(Formula B).

Main compounds in Formula A		
No.	Compounds	(μg/mg tablet)
1	**Psoralenoside**	**4.53± 0.14**
2	Isopsoralenoside	2.96 ± 0.10
3	Naringin	0.239 ±0.007
4	Hesperidin	1.12 ± 0.04
5	Epimedin A	0.221 ± 0.009
6	Epimedin B	0.214 ± 0.008
7	Epimedin C	1.33 ± 0.01
8	Icariin	0.618± 0.018
9	Coryfolin	0.158 ±0.003
10	Corylifolinin	0.126 ± 0.003
11	Baohuoside-I	1.30 ± 0.03
12	Psoralen	0.0552 ± 0.0012

Main compounds in Formula B		
No.	Compounds	(μg/mg granule)
1	Catalpol	1.97 ±0.21
2	Leonuride	0.221 ±0.009
3	Calycosin-7-O-β-D-glucoside	0.183 ±0.003
4	Hyperoside	0.127 ±0.003
5	Acteoside	0.246 ±0.005
6	Formononetin-7-O-β-D glucoside	0.0946 ±0.002
7	Epimedin A	0.472 ±0.010
8	Calycosin	0.167 ±0.011
9	Epimedin B	1.59 ±0.02
10	Epimedin C	3.01 ±0.22
11	**Icariin**	**3.14 ±0.08**
12	Formononetin	0.0943 ±0.0022
13	Astragaloside IV	1.17 ±0.10
14	Astragaloside II	0.447 ±0.003
15	Baohuoside-I	0.964 ±0.033
16	Astragaloside I	0.479 ±0.003

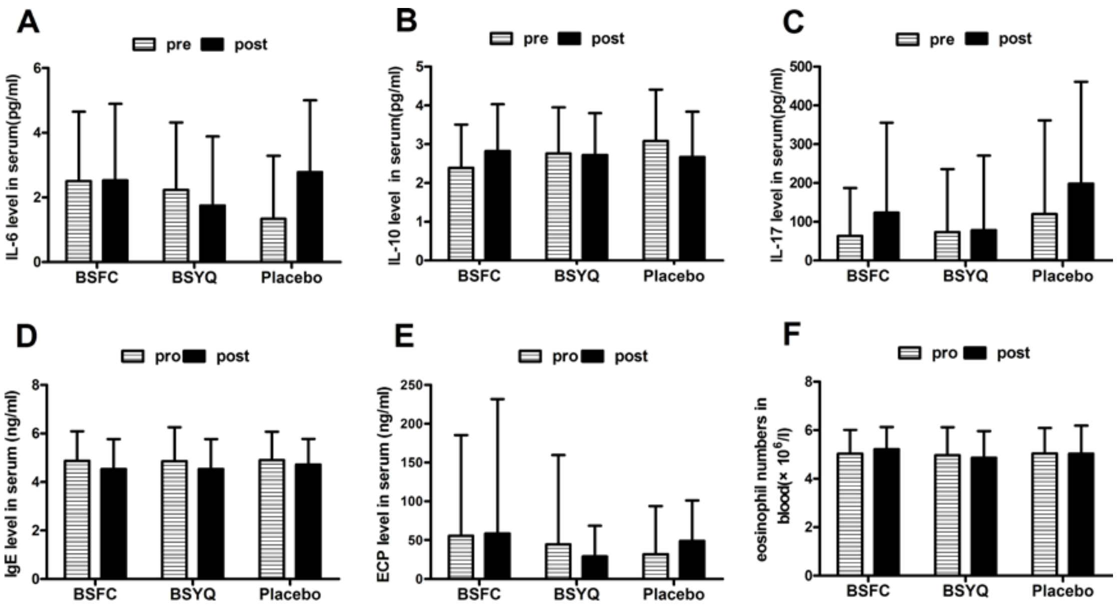

sFigure 2. Effects of 6 months of treatment with two formulae or placebo on serum (A) IL-6, (B)IL-10, (C) IL-17, (D) IgE , (E) ECP and (F) eosinophil numbers. Data are means ±SDs, in addition to IL-17 and ECP (the data were normal distribution), other data were log-transformed. The IL-17, IL-10, IL-6, IgE and ECP were determined by ELISA. For eosinophil numbers count, twenty microliters of peripheral blood collected from a finger stick were diluted in 380 ml staining buffer. The leukocytes were counted by using a hemacytometer. The absolute eosinophil number was calculated. For ELISA measure, venous blood samples were obtained from all patients before and 6 months after treatment. Serum IL-6, IL-10, IL-17, IgE and ECP were measured by commercial ELISA kit (Anogen Yes Biotech Laboratories Ltd, Mississauga, Ontario, Canada and edical & Biological Laboratories Co,.Ltd.Nagoya, Japan) according to the manufacturer’s instructions
